# Supplementary material for: Prevalence of social anxiety disorder and its associated factors among foreign-born undergraduate students in Türkiye: A cross-sectional study
Source: PLOS Glob Public Health. 2024 Jul 30;4(7):e0003184. doi: 10.1371/journal.pgph.0003184 (PMC11288422; doi:10.1371/journal.pgph.0003184)
Supplement: S1 Questionnaire — (DOCX) [file pgph.0003184.s001.docx]

**Prevalence of Social Anxiety Disorder and its associated factors among Foreign-born Undergraduate Students in Türkiye: A Cross-Sectional Study**

Lujain Alnemr^1¶^, Abdelaziz H. Salama^1,2¶*^, Salma Abdelrazek^1&^, Hussein Alfakeer^1&^, Mohamed Ali Alkhateeb^1^, Perihan Torun^3^

^1^ Hamidiye International School of Medicine, University of Health Sciences, Istanbul, Türkiye

^2^ School of Medicine, Johns Hopkins University, Maryland, United States of America

^3^ Department of Public Health, Hamidiye International School of Medicine, University of Health Sciences, Istanbul, Türkiye

**Supplementary file**

**Table 1: Arabic version of Socio-demographic questions**

**Table 2: Arabic version of SPIN Score questions**

**Table 3: English version of Socio-demographic questions**

**Table 4: English version of SPIN score questions**

| **Table 1 : Arabic version of demographic questions**  **قسم 1 / أسئلة اجتماعية – ديموغرافية** |  |
| --- | --- |
| 1. **العمر ؟**   **o 18-21 o 22-25 o 26-29 o 30-33 o 34 and above** | 1. **الجنس ؟**   **o ذكر o أنثى** |
| 1. **الجنسية ؟**   **o تركي الجنسية o غير ذلك (الرجاء الايضاح)** | 1. **الحالة الاجتماعية ؟**   **o غير ذلكo أعزب o متزوج o مطلّق** |
| 1. **عند تقييم دخلك الشهري الإجمالي ، ما هو مستوى دخلك برأيك ؟**   **o منخفض جداًo منخفض o متوسط o فوق المتوسطo عالي** | 1. **هل تدخن ؟**   **o نعم o لا** |
| 1. **هل تشرب الكحول ؟**   **o نعم o لا** | 1. **نوع جامعتك ؟**   **o جامعة حكومية (تابعة للدولة) Devlet**  **o جامعة وقفية (تابعة لجهة غير حكومية) Vakıf**  **o جامعة خاصة (تابعة لشركة أو مؤسسة خاصة ) Özel** |
| 1. **مستوى تعليمك الحالي ؟**   **o طالب جامعي**  **o طالب ماجستير أو دكتوراه**  **o غير ذلك (الرجاء الايضاح)** | 1. **مجال دراستك الحالي ؟**   **o**  **العلوم الاجتماعية (مثال : علم الاجتماع، علم النفس، علم الأنثروبولوجيا، علم السياسة، الاقتصاد).**  **o**  **العلوم الطبيعية (مثال : علم الأحياء، الكيمياء، الفيزياء، علم الجيولوجيا، علم البيئة، علم الفلك).**  **o**  العلوم **الهندسة (مثال : هندسة الميكانيكا، هندسة الكهرباء، هندسة الكيمياء، هندسة الحاسوب، الهندسة المعمارية).**  **o**  **العلوم الصحية (مثال : الطب، التمريض، الصيدلة، طب الأسنان، العلاج الطبيعي).**  **o غير ذلك (الرجاء الايضاح)** |
| 1. **ما هو متوسط ​​درجاتك الأخير ؟**   **o 3.5-4.0 o 3.0-3.5 o 2.5-3.0 o أقل من 2.5** | 1. **ما هو مستواك في اللغة التركية ؟**   **o جيد جداًo جيد o** **معقول o ضعيف** |
| 1. **هل واجهت أي صعوبات في التكيف مع الثقافة التركية ؟**   **o نعم o لا** | 1. **هل تعرضت لأي تمييز أو تحيز بسبب جنسيتك أو خلفيتك العرقية في تركيا ؟**   **o نعم o لا** |
| 1. **هل تشعر بالأمان والأمان عند العيش في تركيا ؟**   **o نعم، دائماً o نعم، في معظم الأحيان o أحيانًا**  **o لا، ليس كثيرًا o لا، أبدًا** | 1. **ما هو أكبر التحديات التي واجهتك كطالب جامعي غير تركي في تركيا ؟**   **o حواجز اللغة**  **o الاختلافات الثقافية**  **o التمييز أو التحيز**  **o صعوبة في العثور على سكن**  **o صعوبة في العثور على عمل**  **o غير ذلك (الرجاء الايضاح)** |

| **Table 2 : Arabic version of SPIC Score questions**  **القسم 2 / مؤشر الرهاب الاجتماعي Social Phobia Inventory ( SPIN )**  *يُرجى قراءة كل عبارة ووضع علامة في الخانة التي تشير إلى مدى تطبيق الموقف عليك خلال* ***الأسبوع الماضي****.* | ***ليس على الإطلاق*** | ***قليلاً جداً*** | ***الى حد ما*** | ***كثير جداّ*** | ***لأقصى حدّ*** |
| --- | --- | --- | --- | --- | --- |
| **1. أشعر بالخوف من الأشخاص ذوي السلطة .** | **0** | **1** | **2** | **3** | **4** |
| **2. . أنا أنزعج عندما يحمر وجهي أمام الناس** | **0** | **1** | **2** | **3** | **4** |
| **3. تخيفني الحفلات والمناسبات الاجتماعيّة .** | **0** | **1** | **2** | **3** | **4** |
| **4. . أتجنّب التحدّث إلى أشخاص لا أعرفهم** | **0** | **1** | **2** | **3** | **4** |
| **5. . يخيفني الانتقاد كثيراً** | **0** | **1** | **2** | **3** | **4** |
| **6. . أتجنّب فعل الأشياء أو التحدّث إلى الناس خوفًا من الإحراج** | **0** | **1** | **2** | **3** | **4** |
| **7. التعرّق أمام الناس يسببّ لي الضيق .** | **0** | **1** | **2** | **3** | **4** |
| **8. أتجنّب الذهاب إلى الحفلات .** | **0** | **1** | **2** | **3** | **4** |
| **9. أتجنّب الأنشطة التي أكون فيها مركز الاهتمام .** | **0** | **1** | **2** | **3** | **4** |
| **10. . التحدّث إلى الغرباء يخيفني** | **0** | **1** | **2** | **3** | **4** |
| **11. . أتجنّب الاضّطرار إلى إلقاء خطاب** | **0** | **1** | **2** | **3** | **4** |
| **12.سأفعل أي شيء لتجنّب الإنتقاد .** | **0** | **1** | **2** | **3** | **4** |
| **13. . يُزعجني خفقان القلب عندما أكون حول الناس** | **0** | **1** | **2** | **3** | **4** |
| **14. أخشى أن أفعل شيئا عندما أكون مراقباً من الناس .** | **0** | **1** | **2** | **3** | **4** |
| **15. . من بين أسوأ مخاوفي أن أكون مُحرجًا أو أن أبدو غبيًا** | **0** | **1** | **2** | **3** | **4** |
| **16. أتجنّب التحدّث إلى أي شخص في السلطة .** | **0** | **1** | **2** | **3** | **4** |
| **17. يُزعجني الارتجاف أو الاهتزاز أمام الآخرين .** | **0** | **1** | **2** | **3** | **4** |

| **Table 3 : Socio-demographic questions**  **Section 1 : Socio-Demographic Questions** |  |
| --- | --- |
|  |  |
| 1. **Age ?**   **o 18-21 o 22-25 o 26-29 o 30-33 o 34 and above** | 1. **Gender ?**   **o Female o Male** |
| 1. **What is your nationality?**   **o Turkish o Other (please specify)** | 1. **Marital status ?**   **o Single o Married o Divorced o Other (please specify)** |
| 1. **When evaluating your total monthly income, what do you consider your income level to be ?**   **o Very low o Low o Average o Above average o High** | 1. **Do you smoke ?**   **o Yes o No** |
| 1. **Do you drink alcohol?**   **o Yes o No** | 1. **What is the type of college ?**   **o Government o Foundation o Private** |
| 1. **What is your current level of education ?**   **o Undergraduate o Graduate o Other (please specify)** | 1. **What is your current level of education ?**   **o Social Sciences (e.g., Sociology, Psychology, Anthropology, Political Science, Economics).**  **o Natural Sciences (e.g., Biology, Chemistry, Physics, Geology, Environmental Science, Astronomy).**  **o Engineering sciences (e.g., Mechanical Engineering, Electrical Engineering, Chemical Engineering, Computer Engineering, architecture).**  **o Medical Sciences (e.g., Medicine, Nursing, Pharmacy, Dentistry, Physical Therapy).**  **o Other (please specify).** |
| 1. **What is your most recent GPA ?**   **o 3.5-4.0 o 3.0-3.5 o 2.5-3.0 o Less than 2.5** | 1. **What is your level of proficiency in the Turkish language ?**   **o Very good o Good o** **Fair o Poor** |
| 1. **Have you experienced any difficulties in adapting to Turkish culture ?**   **o Yes o No** | 1. **Have you encountered any discrimination or prejudice based on your citizenship or ethnic background in Turkey ?**   **o Yes o No** |
| 1. **Do you feel safe and secure living in Turkey ?**   **o Yes, always o Yes, most of the time o Sometimes**  **o No, not very often o No, never** | 1. **What are the biggest challenges you have faced as a non-Turkish university student in Turkey ?**   **o Language barriers**  **o Cultural differences**  **o Discrimination or bias**  **o Difficulty in finding housing**  **o Difficulty in finding employment**  **o Other (please specify)** |

| **Table 4 : SPIN score questions**  **Section 2: Social Phobia Inventory ( SPIN )**  *Please indicate how much the following problems have bothered you during the* ***past week.*** | ***Not at all*** | ***A little bit*** | ***Somewhat*** | ***Very much*** | ***Extremely*** |
| --- | --- | --- | --- | --- | --- |
| **1. I am afraid of people in authority.** | **0** | **1** | **2** | **3** | **4** |
| **2. I am bothered by blushing in front of people.** | **0** | **1** | **2** | **3** | **4** |
| **3. Parties and social events scare me.** | **0** | **1** | **2** | **3** | **4** |
| **4. I avoid talking to people I don’t know.** | **0** | **1** | **2** | **3** | **4** |
| **5. Being criticized scares me a lot.** | **0** | **1** | **2** | **3** | **4** |
| **6. I avoid doing things or speaking to people for fear of embarrassment.** | **0** | **1** | **2** | **3** | **4** |
| **7. Sweating in front of people causes me distress.** | **0** | **1** | **2** | **3** | **4** |
| **8. I avoid going to parties.** | **0** | **1** | **2** | **3** | **4** |
| **9. I avoid activities in which I am the center of attention.** | **0** | **1** | **2** | **3** | **4** |
| **10. Talking to strangers scares me.** | **0** | **1** | **2** | **3** | **4** |
| **11. I avoid having to give speeches.** | **0** | **1** | **2** | **3** | **4** |
| **12. I would do anything to avoid being criticized.** | **0** | **1** | **2** | **3** | **4** |
| **13. Heart palpitations bother me when I am around people.** | **0** | **1** | **2** | **3** | **4** |
| **14. I am afraid of doing things when people might be watching.** | **0** | **1** | **2** | **3** | **4** |
| **15. Being embarrassed or looking stupid are among my worst fears.** | **0** | **1** | **2** | **3** | **4** |
| **16. I avoid speaking to anyone in authority.** | **0** | **1** | **2** | **3** | **4** |
| **17. Trembling or shaking in front of others is distressing to me.** | **0** | **1** | **2** | **3** | **4** |
