# Supplementary material for: Prevalence of social anxiety disorder and its associated factors among foreign-born undergraduate students in Türkiye: A cross-sectional study
Source: PLOS Glob Public Health. 2024 Jul 30;4(7):e0003184. doi: 10.1371/journal.pgph.0003184 (PMC11288422; doi:10.1371/journal.pgph.0003184)
Supplement: Attachment — Submitted filename: SAD PAPER.pdf [file pgph.0003184.s002.pdf]

Minor revisions

Interesting paper

1. Students from countries with civil unrest- Over 60% of the study participants are Syrians- a look at the immigration status could add some interesting information about SAD. Also the remaining study participants from Egypt
2. Monthly budget- Finances- a piece of critical information- how was the range gotten, probably a word or two on this.
3. On Social connection-closeness to classmates is good, but what about access to local community groups - (country-specific) within Turkey?
