# Supplementary material for: Prevalence of social anxiety disorder and its associated factors among foreign-born undergraduate students in Türkiye: A cross-sectional study
Source: PLOS Glob Public Health. 2024 Jul 30;4(7):e0003184. doi: 10.1371/journal.pgph.0003184 (PMC11288422; doi:10.1371/journal.pgph.0003184)
Supplement: Attachment — Submitted filename: Response to Reviewers.docx [file pgph.0003184.s003.docx]

**Response to Reviewers**

Dear Dr. Eboreime,

Thank you for giving us the opportunity to submit a revised draft of the manuscript “Prevalence of Social Anxiety Disorder and its Associated Factors among Foreign-born Undergraduate Students in Türkiye: A Cross-Sectional Study” for publication in PLOS Global Public Health Journal. We appreciate the time and effort that you and the reviewers have dedicated to providing feedback on our manuscript, and we are grateful for the insightful comments and valuable improvements suggested for our paper. We have incorporated most of the suggestions made by the reviewers, and these changes are highlighted within the manuscript. Please see below, in blue, for a point-by-point response to the reviewers’ comments and concerns. All page numbers refer to the revised manuscript file with tracked changes.

**Reviewers' Comments to the Authors:**

**Reviewer #1** Prevalence of Social Anxiety Disorder and its Associated Factors among Foreign-born Undergraduate Students in Türkiye: A Cross-Sectional Study

Abstract

-You need to add keywords

**Author response**: Thank you for pointing this out.

The revised text reads as follows on [line 52]

“[Keyword: Social phobia, Anxiety disorders, Mental health, Social Phobia Inventory.]”

Methods

-Please provide the number of the ethical approval

**Author response**: Thank you for noting this. The [approval number 23/379] has been added on [line 192]

*“[Prior to commencing the cross-sectional study, the research team obtained ethical approval from the Hamidiye Scientific Research Ethics Committee* *(approval number 23/379) [31].]”*

-You need to add more sample size. I don’t think this is sufficient for the study

**Author response**: Thank you for highlighting this aspect. While we acknowledge that a larger sample size can enhance the strength of the study findings, we believe that our current sample size is adequate. Our study initially collected 506 responses, but after screening, we identified 455 eligible participants, which exceeds the minimum required sample size of 255 participants for a population size of 260,316.

Discussion

-Please explain more detail why the students experienced symptoms SAD based on the references (line 279)

**Author response**: We think this is an excellent suggestion. More discussion on this point can be found on [278-285]

*“ [**The high levels of SAD symptoms experienced by students may be attributed to the novel life experiences introduced by university enrollment. These include new living arrangements, forming social relationships, and balancing academic commitments with other responsibilities [15]. In addition, while interpersonal communication and speaking in front of others are crucial skills in students' lives, careers, and academic pursuits, today's college students frequently struggle with interpersonal communication issues, with social anxiety being one of the major psychological issues affecting their academic performance and quality of life [32,33].]”*

-Please do dissection as well with the other factors relating to the presence SAD that not significant

**Author response**: We have added the suggested content to the manuscript on [426-476], where we discuss the students' subject of study, monthly budget, alcohol consumption, and their association with SAD.

*“[**We investigated the prevalence of SAD among students from various academic disciplines, including medicine, engineering, social sciences, and natural sciences. Our findings did not reveal a significant correlation between SAD and the field of study. This contrasts with previous studies, such as one conducted in Ethiopia, which found that SAD was more prevalent among engineering students compared to those studying social sciences and humanities [10]. Additionally, a study conducted among Swedish students reported that SAD was less common among those in pedagogic programs, which primarily attract future teachers [35]. However, research examining the prevalence of SAD across different academic disciplines remains limited. Most studies have focused primarily on medical students, consistently reporting high rates of SAD within this group [14,34,69,70]. Future research should compare the prevalence of SAD across various academic fields rather than concentrating on a single population. Academic success, regardless of the discipline may be more closely associated with SAD than the specific field of study itself.*

*In discussing the relationship between monthly budget and SAD, it is notable that 48.1% of the participants subjectively assessed their monthly budget as average according to a 5-point Likert scale, while 26.8% considered their monthly budget to be low. Interestingly, the analysis did not reveal a significant correlation between the different monthly budget categories and the prevalence of SAD. In a study among Nigerian students by Bella and Omigbodun, the data suggested that students with lower monthly allowances might experience higher rates of SAD [71]. However, in agreement with our results, the analysis showed that this apparent relationship was not strong enough to confidently conclude that lower monthly allowances cause higher rates of SAD. Other studies worldwide have also investigated the prevalence of SAD and its relation to monthly budgets or family income and have shown similar results [10,14,72]. In contrast, one study found that students with higher incomes had less severe SAD [34]. It is reasonable to infer that, since students generally do not work and earn their own income, their lifestyles and financial needs are relatively similar. This homogeneity in their economic conditions may account for the lack of a significant correlation between monthly budget categories and the prevalence of SAD. Alcohol consumption was another factor examined in our study. However, only 11 participants reported occasional use, while the majority reported no use of alcohol. As a result, no significant relationships between alcohol consumption and SAD was found in our results. This trend likely reflects the cultural and religious beliefs prevalent among our participants coming from predominantly Arab and Muslim countries. Previous studies on this topic, however, have yielded contradictory results. For instance, a Canadian study found that 24% of school dropouts had social phobia and high-risk behaviors like alcohol dependence [73]. This can be explained by the fact that individuals tend to use substances like alcohol as a coping mechanism to manage fear, boost self-esteem and improve social confidence as suggested by a meta-analysis by Etkin and Wager [74]. Despite some reports indicating significant associations between SAD and alcohol related problems [75,76], other studies didn’t support a relationship between hazardous alcohol use and SAD [71, 77, 78]. While one study found no significant correlation between SAD and alcohol use [71], another study found that peer dynamics and social networks have a more prominent effect on alcohol consumption than social anxiety [77]. Moreover, in a longitudinal survey of 1,920 adults above 18 years, researchers found that those with subthreshold forms of SAD were more vulnerable to alcohol related problems than those with the full blown disorder [78]. These findings can be attributed to the tendency of students with full blown social phobia to avoid feared social situations and thus not using alcohol as a coping mechanism.]”*

**Reviewer #2** Interesting paper

**Author response**: Thank you!

-Students from countries with civil unrest- Over 60% of the study participants are Syrians- a look at the immigration status could add some interesting information in relationship to SAD. Also for the remaining study participants from Egypt.

Author response: Thank you for this suggestion. We think that is interesting to explore this aspect further. In response, we have incorporated additional information regarding immigration status and its correlation with SAD. Furthermore, we have expanded our discussion on participants originating from Egypt.

The revised text reads as follows on [line 320-338]

*“ [**A great risk of poor psychological health for immigrants has been associated with pressures, such as family conflict, discrimination, and the demands of acculturation [39]. A study conducted in Jordan among Syrian refugee youth aged 15–25 years revealed a high prevalence of SAD, with older youth aged 19–25, who are at the typical university age, being more likely to experience SAD compared to younger individuals, likely due to numerous issues and challenges related to war conflicts and immigration, which can affect their social interactions and relationships, leading to various social and psychological problems [40]. Another study performed in Sudan on long-term internally displaced persons (IDPs) highlighted high prevalence rates of SAD among IDPs, with disability and prolonged displacement significantly increasing the risk, in a country that has experienced one of the worst population displacements in the world due to the world's longest-running civil war [41].*

*Moreover, Egyptian students, constituting the second largest group in our study, represented 11.4% of the population. This observation underscores the ongoing economic challenges faced by the country, as evidenced by the Egyptian pound being rated one of the poorest-performing currencies in 2023[42]. Despite having the financial means to do so, the trend of Egyptian youth choosing to study in countries like Turkey instead of traditional destinations like the US or Europe suggests a desire to distance themselves from Egypt due to perceived future uncertainties [43].] ”*

-Monthly budget- Finances- a piece of critical information- how was the range gotten, probably a word or two on this.

**Author response**: we have endeavored to address the feedback from both reviewers comprehensively. This includes expanding on how the range of monthly budgets was determined, as suggested by reviewer 2. Additionally, in response to reviewer 1’s suggestion, we have discussed factors associated with SAD that did not show statistical significance, such as monthly budget. By integrating these revisions, we believe the manuscript now provides a more thorough exploration of the topic.

The revised text reads as follows on [line 441-456]

*“ [In discussing the relationship between monthly budget and SAD, it is notable that 48.1% of the participants subjectively assessed their monthly budget as average according to a 5-point Likert scale, while 26.8% considered their monthly budget to be low. Interestingly, the analysis did not reveal a significant correlation between the different monthly budget categories and the prevalence of SAD. In a study among Nigerian students by Bella and Omigbodun, the data suggested that students with lower monthly allowances might experience higher rates of SAD [71]. However, in agreement with our results, the analysis showed that this apparent relationship was not strong enough to confidently conclude that lower monthly allowances cause higher rates of SAD. Other studies worldwide have also investigated the prevalence of SAD and its relation to monthly budgets or family income and have shown similar results [10,14,72]. In contrast, one study found that students with higher incomes had less severe SAD [34]. It is reasonable to infer that, since students generally do not work and earn their own income, their lifestyles and financial needs are relatively similar. This homogeneity in their economic conditions may account for the lack of a significant correlation between monthly budget categories and the prevalence of SAD.]”*

-On Social connection- closeness to classmates is good, but what about access to local community groups – (country-specific) within Turkey?

**Author response**: Thank you for highlighting this point. We have addressed the access of foreign-born students to country-specific community groups in Türkiye, as suggested.

The revised text reads as follows on [line 344-348]

*“ [**Indeed, there are numerous local community groups in Türkiye, particularly in major cities like Istanbul which can help students establish a sense of community. However, studying at universities located in these large cities presents significant challenges for students due to the high level of competition. Consequently, many students seek education in urban areas with less diversity in both the population and their peers.]”*
